# Combining a Multi-Agent System and Communication Middleware for Smart Home Control: A Universal Control Platform Architecture

**DOI:** 10.3390/s17092135

**Published:** 2017-09-16

**Authors:** Song Zheng, Qi Zhang, Rong Zheng, Bi-Qin Huang, Yi-Lin Song, Xin-Chu Chen

**Affiliations:** 1 College of Electrical Engineering and Automation, Fuzhou University, Fuzhou 350116, China; n150127048@fzu.edu.cn; 2 Research Institute of Fujian Histron Group Co., Ltd., Fuzhou 350116, China; rzheng2017@histron.cn (R.Z.); huangbiqin2017@histron.cn (B.-Q.H.); songyilin@histron.cn (Y.-L.S.); chenxinchu2017@histron.cn (X.-C.C.); 3 Fujian Provincial Key Laboratory of Industrial Control Information Security Technology, Fuzhou 350116, China

**Keywords:** smart home, universal control platform, collaborative control, multi-agent, heterogeneous devices, communication middleware

## Abstract

In recent years, the smart home field has gained wide attention for its broad application prospects. However, families using smart home systems must usually adopt various heterogeneous smart devices, including sensors and devices, which makes it more difficult to manage and control their home system. How to design a unified control platform to deal with the collaborative control problem of heterogeneous smart devices is one of the greatest challenges in the current smart home field. The main contribution of this paper is to propose a universal smart home control platform architecture (IAPhome) based on a multi-agent system and communication middleware, which shows significant adaptability and advantages in many aspects, including heterogeneous devices connectivity, collaborative control, human-computer interaction and user self-management. The communication middleware is an important foundation to design and implement this architecture which makes it possible to integrate heterogeneous smart devices in a flexible way. A concrete method of applying the multi-agent software technique to solve the integrated control problem of the smart home system is also presented. The proposed platform architecture has been tested in a real smart home environment, and the results indicate that the effectiveness of our approach for solving the collaborative control problem of different smart devices.

## 1. Introduction

The smart home industry is an important field which has strong commercial attractiveness, and is currently in a stage of rapid development, due to the development and application of information and control technologies. According to [1], the growth of the Chinese smart home market is expected to be around 25% from 2012 to 2020, ultimately reaching 357.6 billion yuan in value. Today, Apple, Huawei, Xiaomi, and other IT companies are striving to research and launch smart home platforms based on their own technical standards. In Homekit [2], an iPhone or iPad is used as the family control center, and the smart home applications are built into the iOS operating system. However, its standard is unified, regulated by Apple, and the smart devices’ manufacturers’ products need to be verified by Apple before they are launched. Unlike Apple’s smart home platform, Huawei Openlife [3] adopts an open connection platform, and provides the application programming interface (API) to the public, but it still needs to actively seek multi-cooperation with many device manufacturers, operators, and so on, considering the diversification of smart devices and the communication modes. The Xiaomi MIJIA smart home [4] uses Xiaomi routers as the control center, and allows users to install them autonomously.

Although the entire smart home market has experienced significant growth and related technologies have also accumulated over the past 20 years, the current overall status is still stuck at the level of devices/sensors and network infrastructure development or the platform mode exploration, and has not yet formed a strong market-influential smart home system [1,5,6,7,8,9,10]. The main reason is that with the rise of the Internet of Things industry, more and more types of smart devices are connected to the smart home system, the information coupling strength among them increases, and the home system composition, and communication methods are also becoming more complicated [11,12,13]. At the same time, people are placing higher requirements on the intelligent management strategy of family life, such as how to autonomously increase, adjust, or operate a variety of smart devices with different network protocols, how to bring the heterogeneous devices to function as a team to adapt to the change of application scenes, or meet the individual control needs for people in the future [1,14,15].

An ideal solution for these issues has not yet been found in existing technologies or products, and we believe that the major technical bottlenecks can be summarized as follows: firstly, the interoperability of home devices is limited by their heterogeneity, and there is a lack of a unified technical standard, tool, and algorithm mode to support the development, testing, application, and maintenance of smart home control systems. Secondly, the number of home sensors or devices is increasing continuously, in which case the collaborative control among devices has become more and more important, and also been viewed as a main development direction of the smart home technology in the future, in order to truly meet the personalized needs of each family. These are also where the smart home technology differs from the industrial automation technology of other applying fields currently.

According to these observations, it is necessary to study and develop a universal control platform for smart homes. Thus, this paper proposes a design solution of a universal control platform architecture for the smart home, provides the implementation methods, and verifies its feasibility by experiments. This platform has the following features: it is derived from the industrial control technology field, it supports modular and graphical configurations, adapts to many types of operating system environments, uses a unified linkage and dynamic control or management mode for smart devices, provides adequate software tools and configuration resources, its collaborative control capability can be verified by engineering, and it has the important advantage of a low development cost.

The rest of this paper is structured as follows: Section 2 describes the related work. Section 3 presents a proposed architecture of the universal control platform for smart home (IAPhome) overview. Section 4 details the key technologies and software module implementation. Section 5 describes the implementation examples and results. Finally, Section 6 summarizes the conclusions and presents a future outlook.

## 2. Universal Control Platform

Obviously, how to design a universal control platform for smart home has been regarded as an important challenge. Academia has devoted a significant amount of attention and research to this problem, and has also tried to provide solutions in recent years [1,16,17,18,19,20]. Xu et al. proposed a “software-defined smart home” (SDSH) platform [1]. This provides a network communication technology which can access, or be compatible with, a variety of smart device hardware, and also describes a virtualization technique which is responsible for shielding the communication details, as well as abstracting system resources. Moazzami et al. proposed a smartphone-based platform for smart-home IoT systems (SPOT), which offers a software framework to realize unified APIs for different device vendors and allow device drivers to be defined in and composed by using a novel XML structure [18]. This design can solve the problem of the lack of a unified application interface in human-computer interaction systems due to the usage of heterogeneous home devices, and can also tackle the interoperability of multi-vendor devices to a certain extent. Valero et al. developed a smart home system using the Magentix2 platform, and argued that the smart home system can be viewed as a multi-agent system with distributed agents, and the specifications provided by the multi-agent system can help to adjust and control the system behaviors or services dynamically, in order to meet the personalized needs of family life [19]. Sun et al. proposed a design framework of the multi-agent system for smart home or home automation applications, and presented an agent model for individual behavior, as well as a control method for multi-agent groups [20]. Wei et al. viewed that each entity in the smart home, such as the home network devices, application software, and even the home users themselves, can be associated with a corresponding agent, and the interaction or collaboration among different entities can also be conducted by these agents [21]. In our view, the key issue in developing a smart home system is to build a multi-agent system.

**Figure 1:**
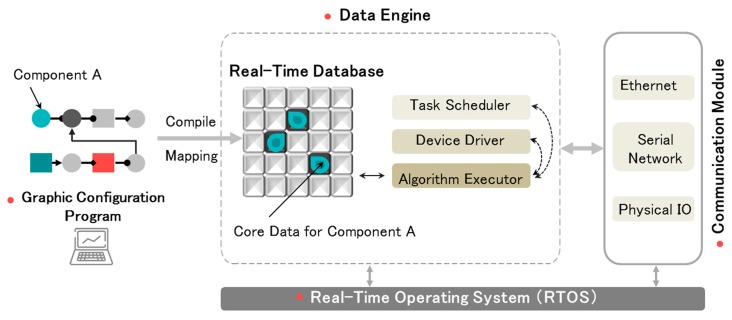
Data Engine scheme.

As to the interoperability of heterogeneous devices, Zheng et al. have presented the industrial automation universal technology platform (IAP) based on Data Engine (DE) technology many years ago [22,23,24,25]. The so-called Data Engine is a middleware software technology based on a multi-agent system, which is used for explaining and processing the controller configuration algorithm. This technology can effectively solve a series of technical problems for the traditional heterogeneous controller, such as how to integrate the multi-PLC heterogeneous controllers into the same platform, or how to perform a unified control configuration algorithm in these controllers [22]. As shown in Figure 1, based on the Data Engine, the control algorithm, submitted as a set of component configurations, can be converted to data streams and mapped to the in-memory database of the control station, and the algorithm’s execution can be driven by updating it [22]. This feature makes the IAP architecture more open and helps it better adapt to connecting a significant number of smart devices, as well as promotes their interoperability, compared to other similar technologies. Additionally, the IAP platform also provides a set of software tools for modular configurations and interactive monitoring, as well as a wealth of control component resources, to flexibly meet the demands of different industries’ control applications. Although this technology was first proposed for the traditional industrial control fields of the electric power and chemical industries, in recent years it has been applied and developed rapidly in many other fields, such as robot control [23], industrial information security [24], ship computing environments [25], and so on. Especially in the Internet of Things (IoT) technology application field, due to the adaptation of the cloud control environment, IAP shows good adaptability in solving some important problems, including interconnection and collaborative control. Therefore, the IAP platform has the technical characteristics of a smart home control platform, as well as the potential to adapt to future industry trends.

This paper first explores the feasibility of applying IAP platform to the smart home field, and proposes a smart home universal control platform architecture (IAPhome) on the basis of IAP technology. This new smart home platform mainly focuses on these technical problems, including the network heterogeneity of smart home system, collaborative control of smart devices, human-computer interaction (HCI), and user self-management.

## 3. Platform Architecture Design

**Figure 2:**
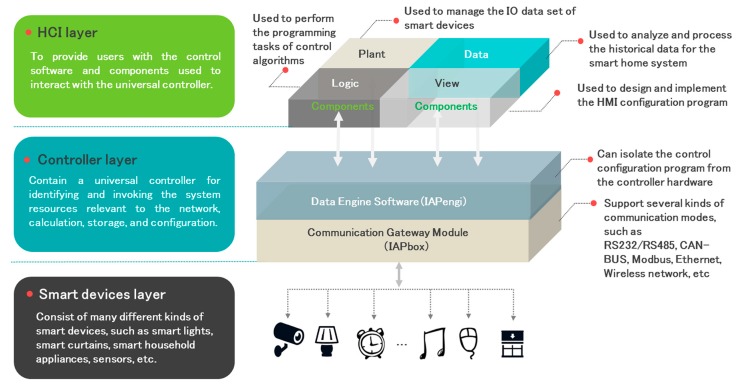
IAPhome software architecture and principle.

The design of the IAPhome platform involves many aspects, such as the universal controller design, the interconnection realization, the programming and execution of control algorithm, and the research of the human-machine interface (HMI). As shown in Figure 2, IAPhome divides the smart home platform into three levels, i.e., the smart device layer, controller layer, and human-computer interaction layer.

The smart device layer consists of a variety of different brands, functions, and models of smart devices, including sensors, smart lights, curtains, home appliances, etc. The controller layer is the core of the entire IAPhome, and its critical part is a universal controller. This controller usually uses IPC as its hardware, and specifically embeds the communication gateway module IAPbox and the Data Engine software IAPengi, which are used for identifying or invoking the system resources relevant to the network, calculation, storage, and configuration. IAPbox is a type of communication middleware which is responsible for connecting and managing a large number of smart devices, and specially developed for solving the problem that different smart devices based on different network protocols cannot communicate with each other. Now it is compatible with RS232/RS485, ONVIF, CAN-BUS, Modbus, Ethernet, wireless networks, and other modes of communication, and IAPengi is used for parsing and processing the control configuration algorithm of the entire smart home system. Since the Data Engine software has been embedded in the universal controller for executing the control configuration, the configuration program of the entire smart home system will be effectively isolated from the bottom of the controller. Thus, from the algorithm execution level of view, the heterogeneity that hinders the interoperability of smart home devices is further eliminated, and the complexity of the overall system control is also reduced. In this way, IAPhome can be compatible with most of the smart home devices and perform a unified standard configuration program.

The software used to interact with the universal controller for users is located at the top of the system, that is, the human-computer interaction (HCI) layer. The available software mainly includes the unified HMI software IAPview, the device-oriented database configuration software IAPplant, the graphical configuration programming tool IAPlogic, and the configuration component library which is encapsulated with specific algorithms (including the artificial intelligence algorithm). In addition, the HCI layer is also equipped with the data analysis software IAPdata for historical data processing, intelligent alarms, performance analysis, and other services, to assist users in a timely manner to grasp the working status information of each sensor or device and observe the working effect of control instructions, as well as analyze the reasons of a variety of alarm information or fault phenomena.

In the system shown in Figure 2, users can design a specific application scene according to the habits or needs of their family life, and autonomously design and modify the control strategy in the interactive software. Then the users’ control data can be sent to the gateway module after being processed by the Data Engine, so as to drive the smart devices to complete the specified operation. In the IAPhome system, all control models of smart devices can be embedded in reusable configuration components, and even the complex control algorithms can be realized only by adjusting the combination of these components. The topology of the control algorithm generated by the components combination will be mapped to the memory of the universal controller and executed by it. The computation sequence of components is determined by the topology sorting algorithm of the directed graph, and it is automatically completed by the configuration software tool at compile time [22,23,24,25]. At the same time, the universal controller can also collect the data of all kinds of smart devices through IAPbox, and send it to IAPengi to complete the data storage, algorithm calculation, and other functions, and finally return the running data of the smart devices to the interactive software for users to realize real-time monitoring.

The overall design and architectural principles of the IAPhome platform are detailed above. However, different home sensors or devices use different types of network protocols, and how does one achieve interoperability among these heterogeneous devices? Application scenes of family life become diversified, and how does one make heterogeneous devices work together in order to adapt to this change? Furthermore, as the demand of people’s individual requirements become more significant, how can IAPhome allow users to autonomously manage, control, and monitor a variety of smart devices? The fact can be found in Section 4.

## 4. Key Technologies and Platform Implement

### 4.1. Smart Home Communication Technology

#### 4.1.1. Types of Communication Protocols

Network communication plays a key role in the smart home control system [26,27], but there exists obvious heterogeneity. Especially, the heterogeneity of different smart devices used as network nodes can have an effect on many issues, including the effective integration of home sensors or devices, and whether different devices can be interoperable.

Table 1 shows the popular communication protocols used in smart homes, such as Zigbee, WiFi, Ethernet, and PLC-BUS [28,29,30,31,32]. In general, wire communication technology has long transmission distance, high speed, and large network capacity, while the wireless communication needs no wiring and has a more flexible network structure with some shortcomings, such as mutual interference and unstable communication. In view of these advantages and disadvantages, currently different types of network protocols cannot be substituted for each other and be widely used in smart home devices.

#### 4.1.2. Network Communication Principle of the Controller

**Figure 3:**
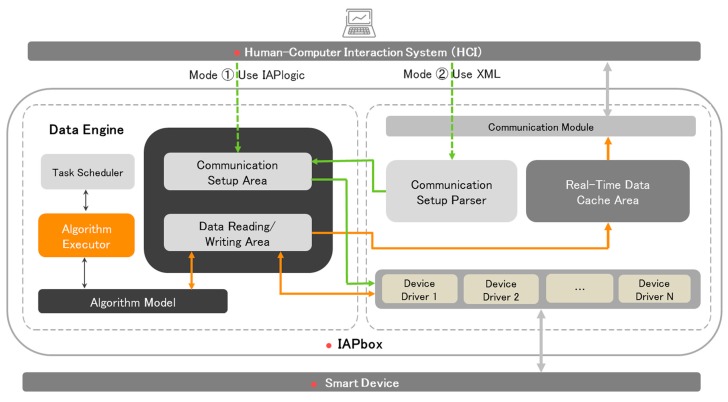
Network communication principle of the controller.

In order to shield the heterogeneity of smart devices in the underlying network and integrate vast amounts of data from diverse sources, IAPhome uses a set of network communication technologies (IAPbox), which is compatible with a variety of communication protocols in the controller layer. As shown in Figure 3, IAPbox embeds a Data Engine unit which differs from other Data Engines by its special usage for storing and processing the communication-related data or arithmetic from the HCI system and smart devices drivers. The basic principle of the IAPbox is to integrate a set of communication drivers into the controller which is usually a computer system based on ARM or x86 architectures, and obtain data from different sensors or devices by invoking the software communication interface API derived from various device manufacturers, and then store it in the specified area of the Data Engine unit in the memory database. With respect to the Data Engine unit, two crucial data areas are designed in its in-memory database, that is, the communication setup data area and the data reading/writing area which are, respectively, used for storing the communication setup data and real-time data of each smart device. Additionally, the Data Engine unit also provides a set of algorithm models, which are specifically used for parsing and processing the initial data of field smart devices and returning results to the data reading/writing area. This algorithm model can be pre-configured in the HCI system, and then downloaded and executed by the algorithm execution module of the Data Engine. In this way, the controller can synchronize data with the field smart devices in real-time, and store it in the corresponding memory area. Since the data of each device have been converted into a unified form for storage in the memory, it can be shared and interacted in a more flexible way.

On the other hand, the HCI system can communicate with the controller through the communication module, and IAPbox can also provide a real-time data cache area for storing the real-time data of devices in time from the data reading/writing area for the HCI system to access. Meanwhile, the engineer station, which is used as the human-computer interaction device for the smart home, provides two kinds of communication setup interfaces for users, that is, the XML file and the configuration programming tool IAPlogic. Using either of them, users can set up the parameters of the communication links connected with the target smart devices (support local and remote setup). These parameters mainly include the communication frame format of the communication objects, the data refresh time of IAPbox, start and stop operation of communication drivers, the engineer station IP address and port number, uploaded packet size, cache data upload cycle, and so on. When the XML interface is used, the communication setup data from the HCI system will be analyzed and processed by the communication setup parser first, and then sent to the communication setup area for device drivers to invoke. If the IAPlogic tool is used, the communication setup data will be stored directly to the communication setup data area after being received by the controller, so as to be accessed by the device drivers.

#### 4.1.3. Configurable Network Communication Mode

**Figure 4:**
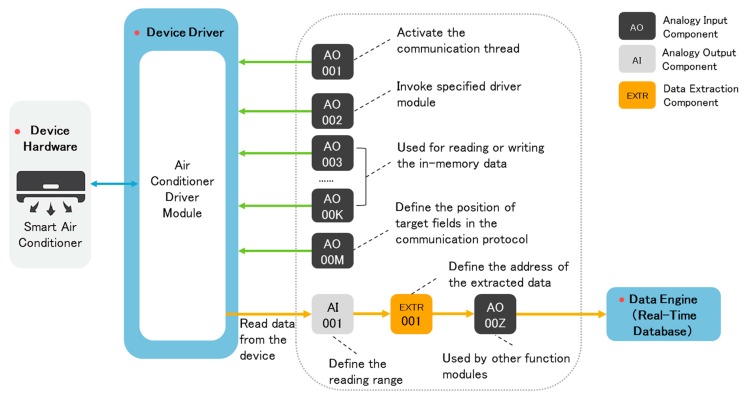
Principle of configurable network communication.

In order to meet the users’ needs for autonomously adjusting and configuring the communication setup data of smart devices, IAPhome adopts a configurable communication management mode, and users can trace the computing intermediate state of components by using the graphic configuration tool (IAPlogic), so as to monitor the activation process of the communication drivers and the operational state of each smart device in a more intuitive and visual way. As shown in Figure 4, the principle of the configurable network communication method is as follows: for the data required or generated by the communication process, users can use the component configuration method to set up communication parameters, such as driver type, gateway device IP, and so on, as well as perform data splitting and splicing. When IAPbox receives these data, the communication connection will be established by assigning the relevant data to the IO points corresponding to the communication parameters. Using components to configure the communication setup data is essential to establish the communication among different hardware by activating their drivers. Moreover, the components also allow users to capture the dynamic data in the process of the drivers’ activation, hardware data reading, and transmission in real-time to realize visual management of the communication process. In particular, when IAPbox deals with the communication of heterogeneous devices, the driver activation process is just to establish the connection of specific network protocol, and perform the packets transmission. In IAPbox, the Data Engine unit can execute a set of algorithm models which are composed of graphical components and used to extract the specific data of communication packets, or to assemble the relevant data into a new communication packet. The data processing of communication packets is implemented by some reusable components. Users just need to edit the component structure in the logic diagrams by the graphical configuration tool, and set specific parameters of each component to complete the device driver processing.

Taking the air-conditioning device of the smart home system as an example, Figure 4 shows an implementation method of how users configure the communication setup data autonomously. As shown in the figure, in order to interact with the smart air conditioner device, we can define the AI and AO components as the data interaction interface, and then activate the communication thread by the communication driver activation component AO 001; use the communication data type component AO 002 to invoke the smart air conditioner driver module; use AO 003–AO 00K to achieve in-memory data reading and writing operations, such as communication parameters, cycles, and other data; and use the communication number configuration component AO 00M to define the number of communication tasks, that is, to obtain the specific position of the target fields in the communication protocol which need to be sent or received. If users want to obtain the working state data of the air-conditioning device, they can use the data reading component AI 001 to define the storage area of the communication data for storing the latest communication packets, then use the data extraction component EXTR 001 to define the specified address of the target data for extracting it from the packets, and finally assign the output value of EXTR 001 to the component AO 00Z for local calculation or further processing by another Data Engine. The way that EXTR extracts the corresponding data depends on the set parameters, which can be configured by users.

#### 4.1.4. Communication Driver Design and Development

In IAPhome controller, we use a thread pool mode to dynamically detect and manage the communication links. The condition monitoring of the underlying sensors or devices and their function control are realized by setting the reader and writer threads, respectively. In many cases, using a multiple Data Engines’ (multi-engine) approach will help to deal with complex control tasks of the smart home system. After the multi-engine boots each time, the thread pool should ensure that the number of threads started is consistent with the number of Data Engine nodes (DE nodes), that is, a thread serves a DE node. At the same time, the system should also be required to invoke two interfaces stored in the form of a DLL, i.e., the multi-engine memory area interface and communication driver interface. Therefore, the users only have to modify the drivers when adjusting the smart device’s access to the home system.

**Figure 5:**
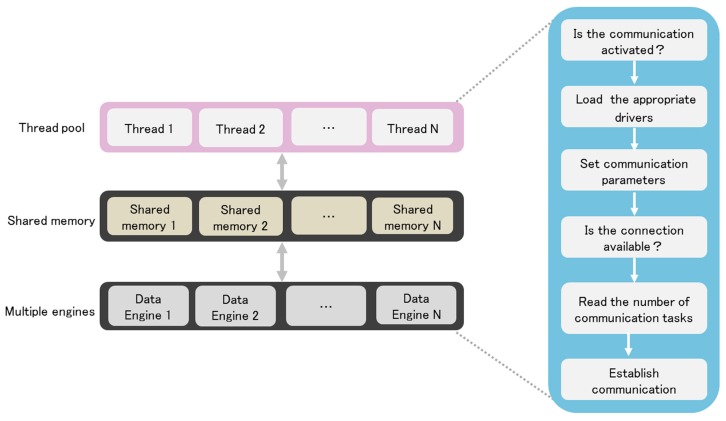
Workflow of the communication driver.

Figure 5 shows the workflow of the communication drivers. As shown in the figure, when the communication driver of a smart device works, the system will first start N DE nodes and create the same amount of shared memory, and then activate N threads in the thread pool and enter a sleep mode, waiting for the engineer station to send the communication data to each DE node. When the sleep thread in the thread pool receives a communication task, it will activate and invoke the corresponding communication driver DLL, and then the communication will be started. Since each DE node is configured with a corresponding Data Engine, the communication logic programmed by users can be loaded and executed by the Data Engine. Due to the above design, the communication gateway software of IAPhome platform studied in this paper can cover a variety of popular communication protocols for smart devices, and can adapt well to the integration needs of a large number of smart devices in the smart home system. At the same time, how to select the home devices need to join the home network, such as smart switches, smart curtains, smart TV and other sensors or devices, can be adjusted based on the user's own requirements and methods, so as to improve the scalability of the entire home control system with regard to different combinations of smart devices in each family.

### 4.2. Multi-Agent Collaborative Control Technology

#### 4.2.1. Multi-Agent Technology of the Smart Home System

The smart home system has significant complexity. Each smart device can be used as an independent functional unit, and should possess the capability of information exchange with other smart devices accessing the family network. More importantly, it can also respond to the demand of users to work together to complete a common task. Therefore, the collaborative control has become one of the most important control modes in the smart home system. The aim to design the universal control platform is to make each smart device work together according to a unified control strategy.

#### 4.2.2. IAPhome Platform Multi-Agent Architecture

**Figure 6:**
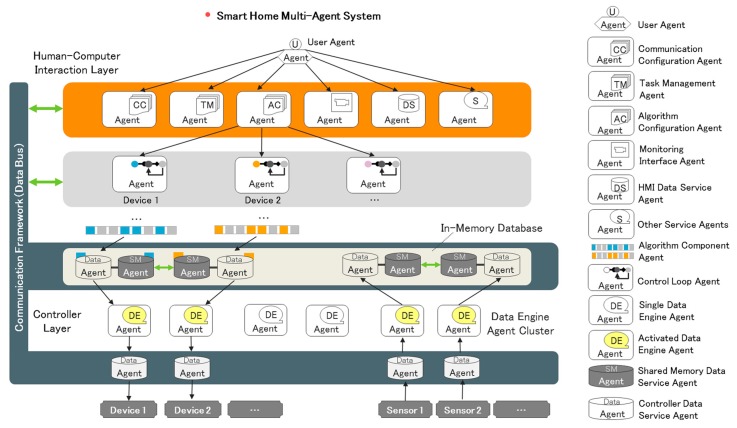
Multi-agent architecture of IAPhome and the information flow of collaborative control.

The IAPhome platform uses a multi-agent system architecture to solve the collaborative control problem of heterogeneous smart devices. In IAPhome, each smart device, functional software, and user entity are associated with a specified agent, respectively, and the collaborative control among multi-devices is essentially implemented by the interaction of these associated agents. As shown in Figure 6, the principle of the IAPhome multi-agent architecture is as follows:

• Human-Computer Interaction Layer

A special agent called a component is designed in the HCI layer, which is also the smallest unit in the multi-agent system. As discussed in Section 3, each component is encapsulated with a specific algorithm which is responsible for implementing the specified functions. These functions can be the device-oriented control instructions according to the user's individualized requirements, or the communication instruction for smart devices, or the management instruction used for task planning, decomposition, and coordination of the entire smart home system. Further, the component agent also has a mobile feature that can release its internal algorithm in the form of data into the in-memory database of the Data Engine in the controller layer, which can make the control data exchange between different devices possible. Additionally, in the HCI layer, the control loop composed of multiple components can be designed as the higher-level agent for each component, and it is responsible for parsing the user's control task requirements, defining the collaborative process of component agents from the functional perspective, and determining the computation sequence of each component invoked by the controller layer agents.

• Controller Layer

The controller layer defines the Data Engine as a virtual agent, which is used to shield the operational details of the universal controller, and to abstract and schedule the system resources of storage, calculation, and communication. The Data Engine is also a kind of multi-agent software organization, and it contains a series of service agents for task execution, resource scheduling, and human-computer interaction. The role of the component algorithm execution agent is particularly important, and when it is in the active state, the data update task corresponding to the component internal algorithm will be driven and performed. In addition, a number of virtual agents can also form an agent cluster, and commonly accomplish the complex control algorithm task that is delivered by the control loop agents and component agents, to improve the resource utilization of the controller.

• Communication Framework

A communication framework has also been designed in the IAPhome multi-agent system to provide a data bus for data exchange and to share among different agents. First of all, a unified data structure is designed in the in-memory database of the Data Engine, and the continuous update of its internal data is accomplished by changing the real-time value and the update time of arrays associated with specified component agents. Additionally, the shared memory region is also established to provide a data exchange and shared channel for different component agents. Secondly, the agents between the human-computer interaction layer and the controller layer can also establish communication through the network bus (such as TCP/IP), and the communication among different agents is realized by reading and writing objects to the specified memory area. In addition, the communication between the controller kernel and the smart devices is realized through the gateway module, and the communication contents mainly include the original communication data from the sensors or devices. And these data can be mirrored in the in-memory database of the controller. Due to this mirroring mode, the process corresponding to each DE node in the controller only needs access to the communication frame to obtain the real-time data of each smart device.

#### 4.2.3. Collaborative Control Based on a Multi-Agent System

Through the design of the multi-agent architecture above, IAPhome can effectively reduce the complexity of the smart home control system, improve the coordination, interactivity and flexibility of the system, and achieve the data exchange and collaborative control among all kinds of home devices/sensors.

As shown in Figure 6, when users demand a collaborative control task of different smart devices, IAPhome can design several types of agents to adapt to it. For example, in order to establish a network connection for different devices or sensors, a set of communication component agents can be designed to set up communication parameters for these smart devices needed to join the smart home network. Then, the control loop agents can be designed to process each control sub-task after the general control task of the system is planned and decomposed. This process can be handled by the graphical configuration based on many component agents. Then, the data content carried out by control loop agents or collected from sensors will be sent to the universal controller through the communication framework and processed by the corresponding Data Engine agents. Finally, the Data Engine agents will send their running data to the corresponding smart devices through the communication framework, and then drive the target devices to complete the specified control functions, so as to achieve the goal of collaborative control.

#### 4.2.4. Human-Computer Interaction in a Multi-Agent System

Another widespread concerning issue of multi-agent collaborative control is the triggering and identifying problem of the scene modes, which refers to the human-computer interaction technology of smart home platforms [33]. At present, most of the smart home control systems have brought in the graphical interface for user interaction controls [34,35,36], for example, Shastri et al. designed a kind of Android mobile client and its human-machine interface for smart home systems [36]. However, the current human-computer interaction interfaces mainly use a blocked strategy based on scene modes, device management, and user profile, and the family users can only use a few simple function keys to achieve smart control, which is difficult to adapt to the differentiated changing needs for different families, and also cannot really achieve the feedback control of interaction interfaces for the entire smart home system [37,38].

**Figure 7:**
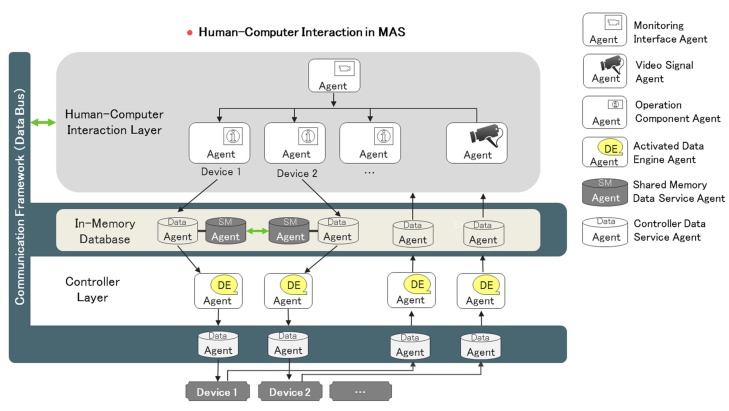
Human-computer interaction in the multi-agent system.

Unlike other control platforms, IAPhome’s human-machine interface provides the overall layout of the entire smart home environment, and each smart device running status and the interoperation process among these devices can be monitored by users. With respect to this feature, the IAPhome human-computer interaction technology plays an important role. As shown in Figure 7, the human-computer interaction environment can provide each driver or each group of drivers with the corresponding operation component agents, in order to make family users directly intervene in the working process of smart devices. The operation component agents, controlled by users, can simultaneously access the corresponding smart devices and their surrounding video signal. All the data in the human-computer interaction system are collected and stored in the controller's memory database, and the human-computer interaction terminal devices can communicate with the data service agents in the controller layer to dynamically obtain the control process data of smart devices in real-time and display it in the HMI interface. In this way, IAPhome truly realizes the visual management and control of home life.

## 5. Case Study and Verification

### 5.1. Smart Home Lab Design

**Figure 8:**
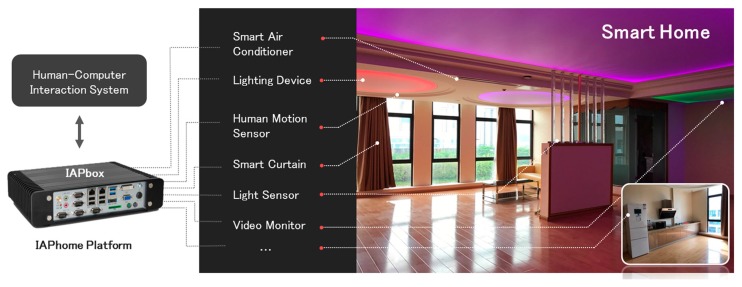
Smart home lab environment.

The IAPhome platform architecture presented in this paper has been implemented in the 85 m^2^ size smart home laboratory environment provided by our research team. As shown in Figure 8, the laboratory is equipped with various sensors, such as human motion sensors, lighting sensors, and smoke sensors, as well as many devices, such as lights (including strip lights, RGB lights, and overhead lights), smart curtains, video surveillance devices, a smart mirror, and a variety of other smart appliances (including air conditioners, a smart refrigerator, and so on). CLEVEROOM brand is selected for the hardware devices of lighting sensors and the human motion sensor. The lighting sensors include both indoor and outdoor types and are used for collecting the illuminance data, and the human motion sensor is installed in the living room to collect human position data. WEIZHILINGT and OPPLE brand lighting are used as the lighting devices. LANBOO brand curtains are selected as smart curtains in the living room. HIKVISION cameras are used as the video surveillance devices and the CVTE smart mirror is used for smart life services, such as weather forecast and music center, as well as a conventional mirror. More details of these devices are provided in Table A1 (Appendix 1). Based on the architecture of the IAPhome platform and its key technologies, this paper develops a set of smart home control systems, and performs the control experiments in several kinds of home smart devices described above. The NORCO BIS-6660D IPC is selected as the universal controller hardware, and the ThinkCentre M8600Q-N000 desktop and Android mobile phones (such as NUBIA NX512J) are used as the engineer station (HCI device). To ensure the reliability and stability of communication effects, the platform uses wired Ethernet communication for smart devices. The communication cycle is set as 50 ms after each smart device is connected to the system. Once the instruction execution time exceeds 50 ms, the system will proceed into the next period; if not, it will proceed into sleep mode until the time reaches 50 ms.

### 5.2. Control Function Modeling

The key to solving the problem of collaborative control of different devices in the smart home system is to provide the control algorithm of each type of device in the controller with the ability to recognize each other. IAPhome provides a method for the mutual recognition of different smart devices. It set up a general model to solve the collaborative control problems of different devices, and describes the model in a unified standard configuration language, and then executes the configuration language to achieve the expected control objectives.

Before that, this paper divides the smart home controls into three types of models. First of all, we set the controlled object as A, the execution instruction as B, the control effect as C, the external condition as D, among which A, B, C and D are corresponding to the set of their ranges. Then each model is defined as follows:

Mode 1: Impose control instructions to the controlled object directly, and generate the corresponding control effect. As to the specified smart device A_i_, the instruction execution state B_j_ can be classified into four types. The control model can be described as in Equation (1):

(1) $$C\left(A_{i}\right)=\{\begin{array}{c} {0,\;\mathrm{Stop}\;B}_{1}\;\\ {1,\;\mathrm{Start}\;B}_{2}\\ {2,\;\mathrm{Device}\;\mathrm{function}\;B}_{3}\\ {4,\;\mathrm{no}\;\mathrm{operation}\;B}_{4} \end{array}$$

Mode 2: Execute the control instruction and generate the corresponding control effect, when there is an external condition and the controlled object is explicit. For all kinds of scene modes Y_k_∈ B, the control model can be described as in Equation (2), where n is the number of smart devices:

(2) $${C(Y}_{k}{)=B(Y}_{k})\cdot \overset{n}{\underset{i=1}{\cup }}{C(A}_{i}),{\;D(Y}_{k}),$$

Mode 3: When the external condition is satisfied and the controlled object is explicit, the corresponding control effect will be automatically generated. For each triggering event X_z_∈B , the control model can be described as in Equation (3), where n is the number of smart devices:

(3) $${C(X}_{z})=\overset{n}{\underset{i=1}{\cup }}{C(A}_{i}),{\;D(X}_{z}),$$

### 5.3. Case Design

In this study, two cases are set up to verify the feasibility of the IAPhome platform architecture, the collaborative control performance of several smart devices, and the human-computer interaction ability.

• Case 1: Collaborative Control Experiment of Smart Devices

In this experiment, the collaborative control effect of the curtains, strip lights, and three kinds of sensors based on the IAPhome platform is studied under the condition of different illuminance and human position data. The control requirements of users are set as follows: in the non-off-home, non-sleep, non-audio, and non-video scene mode, when the indoor illuminance is less than 170 Lux, the outdoor illuminance is less than 660 Lux and the living room human motion sensor detects someone, the strip lights in the living room should be turned on automatically and the curtains will also be closed cooperatively.

• Case 2: Control Experiment in a Specified Scene Mode

The control effect of a specified home life scene and the adaptability of the IAPhome platform architecture is observed in this experiment. The experiment workflow is as follows: For all scenes, set up multiple control nodes for each scene first, and when the specific scene instruction is selected, the control function corresponding to each control node should be executed immediately. Secondly, the mutual exclusion function is set among different scenes. Each time, users can only choose one scene mode and then other modes will be released automatically, in order to prevent an endless loop or random code by operating several scenes at the same time. Finally, keeping the executing state of the current scene, and providing users with an intuitive experience. In the following, a “morning scene” mode is presented as an example, and the experiment workflow is described:

Step 1: Start morning scene, and stop other scenes;
Step 2: Open the living room curtains, and turn on the bedroom RGB light;
Step 3: Human motion sensor detects whether people get up; and
Step 4: Turn down the RGB light and turn on the overhead light and the smart mirror switch.

### 5.4. Results

**Figure 9:**
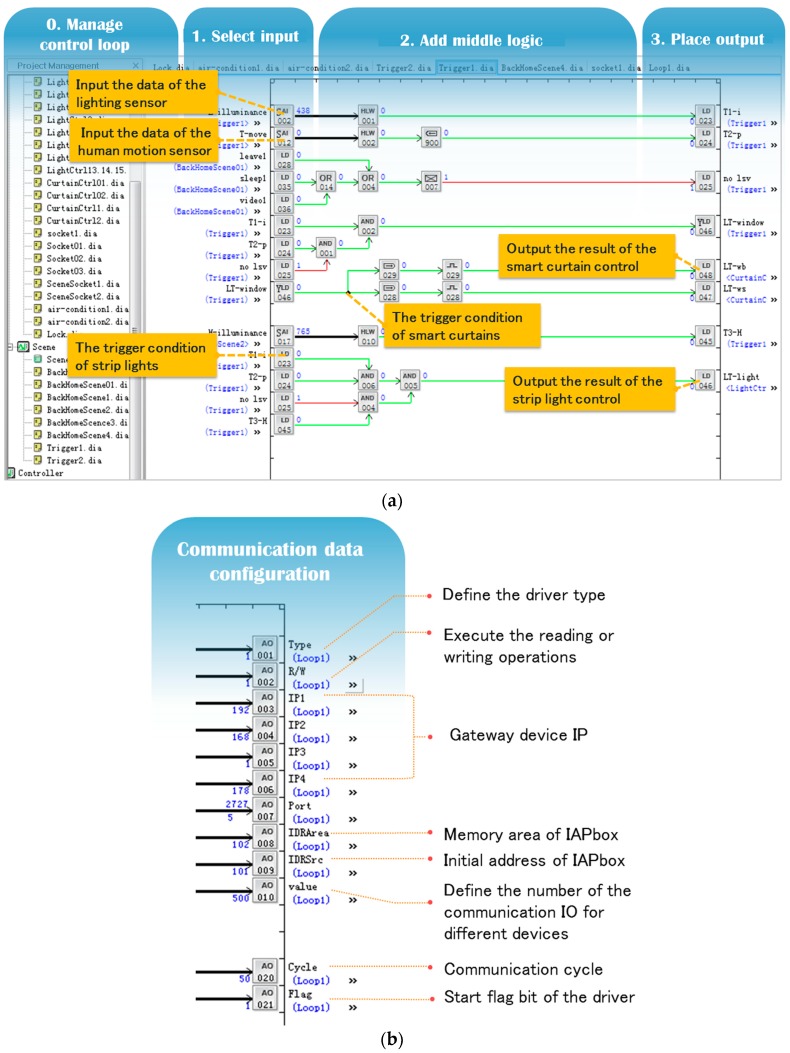

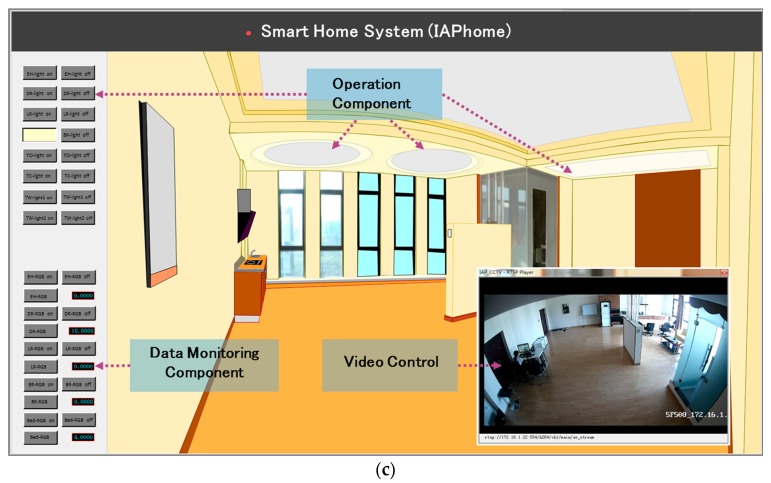
Graphical configuration representation of Case 1. (**a**) Partial program of the algorithmic control logic; (**b**) partial program of the communication setup logic; and (**c**) partial program of the human-machine interface program.

**Figure 10:**
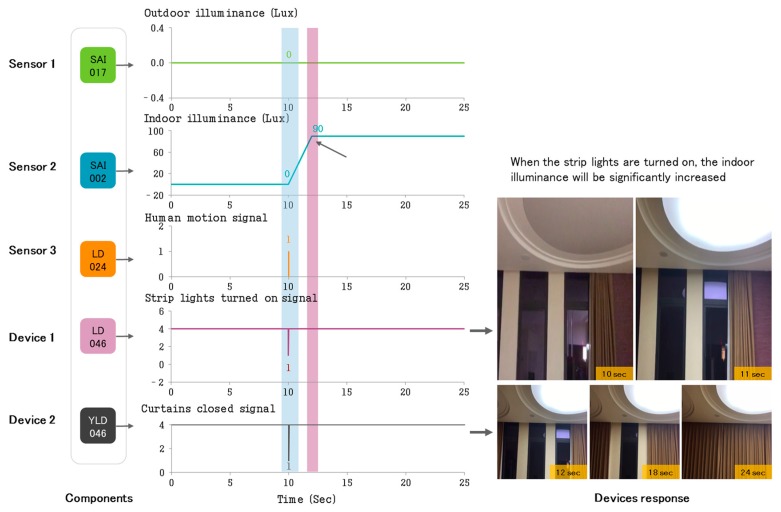
Collaborative control process of multiple sensors and devices.

The experimental results are shown in Figure 9 and Figure 10. Figure 9a–c presents the algorithmic control logic, communication setup logic, and human-machine interface designed for Case 1. As seen in Figure 9a,b, the collaborative control algorithm logic and the communication setup logic of devices are set up by the graphical configuration language. From the figure, we can see that the two types of logic functions (both the control function and the communication function) can be realized by the simple combination of configuration components, and the intermediate state of each component calculation process can also be captured and presented by the monitoring software for users intuitively and transparently. Especially for the communication setup logic, the users only need to simply set up parameters of several components, including the communication driver type, communication address, communication period, and the number of communication IOs, so as to autonomously and flexibly establish the communication connection for each smart device accessing the family network, and monitor the communication status of each device in real-time.

Figure 9c shows the human-machine interface for the overall layout of the entire smart home system. In the figure, some HMI operation components designed for smart devices are presented. All the calculation process data of operation components are recorded in the database in real-time, and the relevant execution records can also be fed back to the monitoring interface. At the same time, by the operation components, users can also invoke the corresponding video signal of each smart device to hold its working status intuitively.

In Case 1, we use Equation (1) to set up the control signal of curtains and strip lights, and use Equation (3) for the triggering of control events. Figure 10 shows the dynamic control process of the lighting sensors, the human motion sensor, the lights, and curtains in the designated collaborative control scheme. In Figure 10, we can see that when the lighting sensors detect that the outdoor illuminance is less than 660 Lux and indoor illuminance is less than 170 Lux, and the human motion sensor detects that the user is at home (human motion signal) at the same time, the living room lights will be automatically turned on, which can significantly increase the indoor illuminance, and the curtains will be closed collaboratively as well.

**Figure 11:**
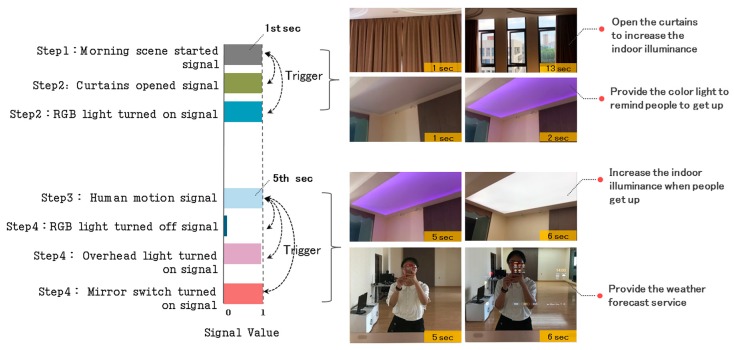
Dynamic control process in the morning scene mode.

In Case 2, we use Equation (1) to set up the control signal of the RGB light, overhead light, and mirror switch, use Equation (2) for the whole scene control, and use Equation (3) to describe the triggering of the relevant control events. Figure 11 shows the dynamic control process of the system in the morning scene.

In Figure 11, when the morning scene mode is started, the system will automatically trigger and open the living room curtains, and turn on the bedroom RGB light to increase the indoor illuminance and remind users to get up. If the human motion sensor detects that the users are getting up (human motion signal), the system will automatically turn off the bedroom RGB light and turn on the overhead light to further increase the indoor illuminance. Additionally, the smart mirror switch will also be turned on collaboratively to provide a weather forecast service for users.

**Figure 12:**
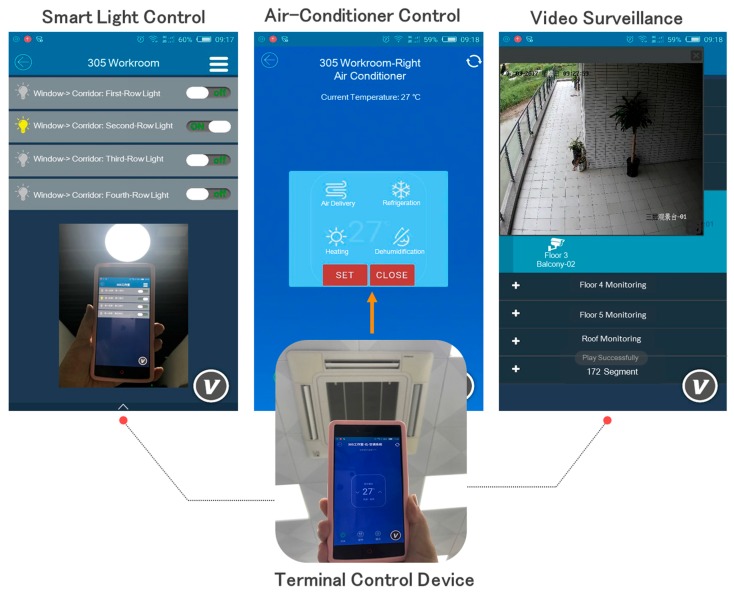
User experience for multi-device control.

In this paper, the Android-based HMI software (V) of IAPhome is also implemented, and some experimenters are invited to test the human-computer interaction function independently. The wireless network (such as WIFI/3G/4G) can be used to establish the connection between experimenters’ mobile devices and the IAPhome controller. An example, using the HMI software for the multi-device control is shown in Figure 12. From the figure, we can observe the running interface and control effect of the air-conditioning system (such as temperature control and pattern regulation), lighting system in the corridor, and the video monitor on the balcony in our smart home laboratory. The results indicate that IAPhome can integrate multi-vendor heterogeneous devices into a unified interactive platform, and use the same application to achieve the device control and interoperate. It should be noted that the implementation of the HMI monitoring function in Figure 12 depends on the function and interaction of the relevant agents, especially the component agents and control loop agents of the graphical logic configuration (such as Figure 9a), and the Data Engine agents in the controller. The real-time data returned by these agents can be dynamically updated to the experimenters’ HMI interactive interface. In this way, IAPhome can not only accurately respond to the experimenters’ control request, but also provide them with an intuitive, good human-computer interaction experience.

## 6. Evaluation and Discussion

IAPhome is a novel universal control platform for smart home. Its universality mainly reflects in two aspects. First, heterogeneous devices can be connected to the gateway module (IAPbox). Second, users can use a unified, graphical control configuration method to achieve the automatic or intelligent control in more modes, including more complicated collaborative control of heterogeneous devices.

• Architectural comparison with other systems

To demonstrate the flexibility and extensibility of our approach, in this section, we compare IAPhome with several existing systems from architecture viewpoint and the features. As shown in Table 2, many platforms, such as HomeKit, can support multi-vendor smart devices of multiple types, and most of them support of devices with local APIs and cloud-based APIs [18]. The difference can be found in terms of third-party extensibility of device support and the flexibility. When adding a new device to the home network, some platforms (such as AllJoyn) only allow the device vendors to develop the drivers, while others allow third parties (with some programming expertise), such as device vendors and integration service providers, to implement the device drivers. SPOT provides an XML-based, unified and relatively simple way to define the drivers to improve the extensibility of smart devices. However, there are some shortcomings, in particular, XML users still need some programming knowledge, and some conditions for SPOT commercial applications in the future can also be found, that is, the vendors or others should actively implement and distribute the drivers that conform to the SPOT specifications. There is still a large uncertainty that whether these conditions can be met.

Compared with some advanced smart home platforms (such as SPOT), IAPhome has several similar features of homogeneous devices integration, for example, the device drivers are developed based on specific APIs. However, IAPhome has some significant features. First, IAPhome uses the unified, graphical software for the reading/writing process of device drivers. The graphical configuration is relatively intuitive, and its data processing process is transparent, that is, the data processed can be visually presented in the software interface (as shown in Figure 9b), and has a dynamic reconfigurable characteristic [22]. In IAPhome, the device network interface can be set up by the configuration software tool or described in XML files, while the decomposition and integration of data packets can be described in the graphical configuration files. Other platforms or technologies that support third-party drivers usually only allow them to conform to specific language specifications such as C#, Java, and XML, which require a relatively higher level of technical expertise, and it is difficult to achieve the transparency of the drivers' executing process, and need higher debugging costs. Second, IAP platform does not demand device vendors to develop the APIs following the "IAP specification", and only requires them to provide APIs with standard network protocols such as RS485, Modbus, and so on. For the ultimate users, the programming details of drivers will be shielded by IAPhome. As described in Section 4.1.3, users only need to use many reusable graphical components to realize the communication management of smart devices, which is more simple and intuitive than other methods. Since all the graphical components can be reused, the internal parameters of the components can be adjusted and the connection between the components can also be changed, the extendibility of heterogeneous devices and the flexibility of IAPhome are more significant. This approach can be particularly effective when the device vendors are reluctant to develop the drivers following others’ specifications.

**Figure 13:**
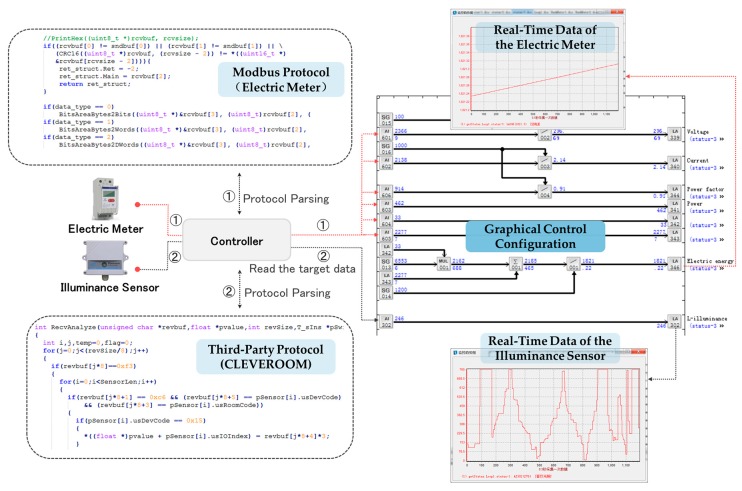
An example of monitoring heterogeneous sensors.

Figure 13 further shows an example of monitoring two actual sensors, including a lighting sensor with the third-party network protocol provided by CLEVEROOM and a smart electric meter based on the Modbus protocol. As shown in the figure, in IAPhome, the gateway module of the controller (IAPbox) can be used to parse the communication protocols of these two heterogeneous sensors respectively, and store the target data into the specified in-memory area. And then we can use a set of AI components in the engineer station to read the target communication data into a same graphical control configuration program, so as to realize the dynamic monitoring of the heterogeneous sensors. From the monitoring trend chart, we can clearly observe that with the changes of external conditions, including the changes in lighting conditions and the household electricity, the real-time data of both the two sensors vary dynamically in the same period.

• Platform Positioning and Indicator

IAPhome adopts part of the industrial control technologies, and its products are focused on the universal control platform, which provides a good solution to the heterogeneous devices communication and the collaborative control problems. Other platforms (such as SPOT) that can solve the heterogeneity of smart home system usually provide users with the unified API and mobile terminal software. The characteristics of the IAPhome control platform, including the control configuration design, the device status monitoring and the collaborative control, have been verified by the cases studied in this paper.

Although these cases just refer to the control of several types of sensors and devices, in the IAPhome system, we can take the same technical standards, tools, and methods as used in these cases to deal with the similar control problems for the more complicated home system with a larger number of different devices. The main difference is the resource usage of the IAPhome platform, such as the number of controllers, the number of Data Engines, the type and number of components, and so on. Table 3 shows several technical indicators of IAPhome. As shown in Table 3, we can see that IAPhome can provide 20-200 Data Engine agents and handle hundreds of home devices (within 4000 IO points) per controller. The communication cycle of each device is not more than 50 ms. And the number of controllers can also be extended according to the users' request. In IAPhome, whether the capacity performance indicators (such as the number of Data Engines and communication IO points) or performance indicators (including the device communication cycle and the types of network protocols supported) are able to meet the control needs of a general smart home system well. Specifically, it also has good extensibility, and the platform resources can be adjusted dynamically based on the scale of each family system and the complexity of its control needs.

**Figure 14:**
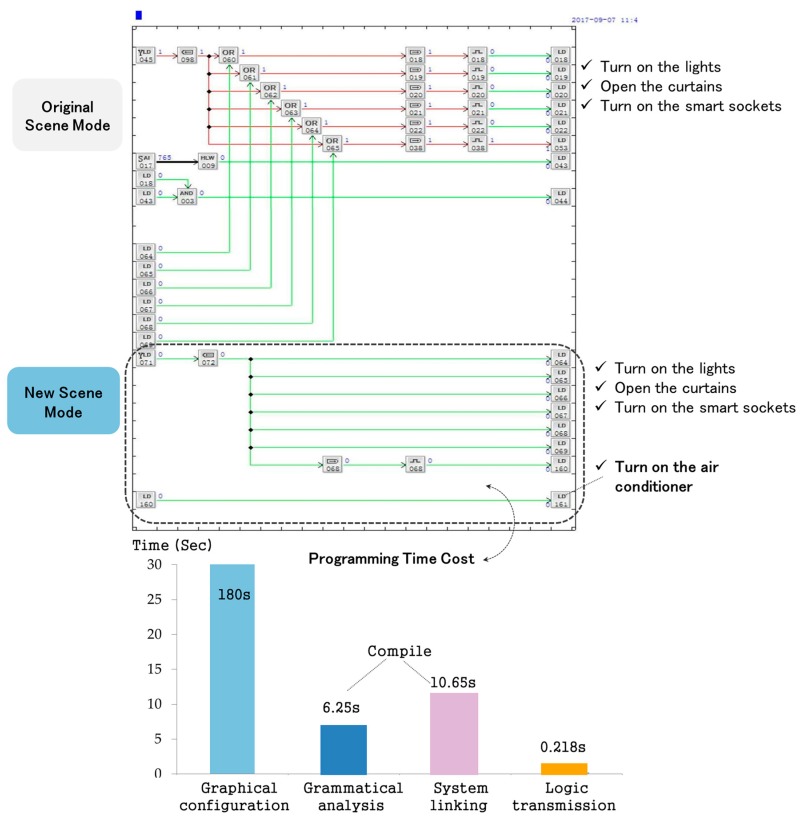
An example of the scene mode extension.

Figure 14 shows a part of the function and performance of IAPhome. As shown in the figure, the original smart home system sets up a “home mode”, which enables the system to automatically turn on multiple lighting devices, curtains and smart sockets when the host gets home. If the user wants the system to open the air conditioner automatically as well as perform the original operations, and make it as a new and optional mode, then it can be easily realized by using a few configuration components in the graphical configuration software. The general users without a lot of computer profession expertise can complete the configuration program for this new function within 3 min. The system compilation time is less than 17 s, including the grammatical analysis time and system linking time, and the time for the control logic to be transmitted to the controller does not exceed 0.3 s. The time cost of the system compile task and logic transmission mainly depends on the number of components used in the current smart home system (1051 in total). In the IAPhome, each controller can process less than 16,000 components during each execution period, and the number of controllers can be expanded continuously. Therefore, this platform can be well adapted to the control needs of general smart home systems and even more complicated control requirements. It can be seen that this method has many advantages, such as easy to operate and debug, high efficient programming process, transparent calculating process, and good extensibility.

## 7. Conclusions and Future Works

The collaborative control of heterogeneous devices is a critical challenge in a smart home control system, and the development of universal control platforms has been viewed as an important way to solve it. The main contribution of this paper is to propose a smart home universal control platform architecture (IAPhome) based on the multi-agent system and communication middleware. IAPhome network communication technology is an important foundation to develop the smart home control system, which establishes the channel of data interaction for heterogeneous devices. The configurable network communication mode provided in this platform helps users adjust their home sensors or devices more autonomously and flexibly. The multi-agent software technology is a crucial part to develop the smart home system based on IAPhome platform.

The configuration components, Data Engine, and communication middleware are the core elements of this platform architecture, which allow each agent in the smart home system to communicate and work with each other to complete a general control task collaboratively, in order to meet the personalized control needs of family users. The results indicate that the application of IAPhome architecture in the smart home system is a scientific and feasible solution. It can effectively solve the heterogeneity and diversity problem of different smart devices, sense the living environment changes in time, make smart devices perform collaborative control, and respond to the family life scenes accurately. In addition, by making the heterogeneous characteristics of smart devices graphical and transparent, IAPhome can reduce the difficulty in developing the home system, and improve the flexibility of the system management for users, as well as adapt well to the personalized control needs of family users. Based on the above characteristics, the IAPhome platform shows significant adaptability and advantages in the connection of heterogeneous devices, collaborative control, human-computer interaction and user self-management.

Smart home technology development has reached an important turning point, and the universal control platform has also entered a rapid development period. The distance between the current smart home technical status and its real goal can be shortened by the universal control platform because it is independent of controllers. The core part of the IAPhome universal control function is derived from the industrial control field and, in this paper, we introduce the network communication technology and improve the multi-agent technology to it, providing a good environment for rapid development in the smart home field. With the application scenes changing more flexibly and the system control becoming more complicated, the IAPhome platform will face new challenges in cloud control, big data applications, artificial intelligence systems, home privacy protection, third-party service, and the business model establishment in the future, but will establish new opportunities as well. Our future plans include extending the smart home system to support more diverse smart devices, and further evaluating the platform by more complicated control applications. In addition, we will further explore the possibilities that whether IAPhome can be applied to other smart home domains, such as more intelligent services depending on the individuals' behavior and the smart home energy management applications.

## Figures and Tables

**Table 1:** Comparison of typical communication methods in smart home.

	IEEE 802.15.4 (Zigbee)	IEEE 802.11 (WiFi)	IEEE 802.3 (Ethernet)	X-10PLC-BUS
Typical transmission distance (m)	5–100	50–300	100	200/X-10 2000/PLC
Network structure	Dynamic routing ad hoc networks	Cellular networks	Star networks	Bus, Star networks
Communication rate (bps)	250,000	1 M–600 M	10 M–1000 M	2M/X-10 200/PLC
Network capacity	255, can be expanded to 65,000	50, depending on AP performance	The segment type determines its capacity, can be unlimited expansion	256 address codes/X-10, 64,000/PLC
Protocol specification	International level IEEE 802.15.4	International level IEEE 802.11	International level TCP/IP	Industrial level

**Table 2:** Comparison of different smart home frameworks. ^1^

Framework	Multi-Vendor	Multi-Type	Support of Devices with Local API	Support of Devices with Cloud-Based API	Third-Party Extensibility ^2^	Unified Interface ^3^
HomeOS	√	√	√	-	√(C#)	-
openHAB	√	√	√	√	√(Java)	-
IFTTT	√	√	-	√	-	-
Nest thermostat	-	-	-		-	-
AllJoyn	√	√	√	√	-	-
HomeKit	√	√	√	√	-	-
Smart Things	√	√	√	√	-	-
SPOT	√	√	√	√	√(XML)	√(API)
IAPhome	√	√	√	√	√(XML/Graphical Tools)	√(Control Platform)

^1^ All data in this table are derived from Reference [18] except IAPhome. ^2^ Third-party Extensibility refers to whether device support can be added parties other than device vendors or service providers [18]. ^3^ Unified Interface refers to whether a technology offers the unified interface to monitor and control each device [18].

**Table 3:** IAPhome platform technical indicators.

Indicator Type	IAPhome Platform
Number of components	≤16,000/controller, the number of controllers can be extended
Number of Data Engines	Any, depending on the hardware performance, usually 20 to 200 per controller
Number of communication IO point	≤4000/controller, the number of controllers can be extended according to the scale of home system
Device communication cycle	≤50 ms
Types of network protocols supported	> 9 types, including RS232/RS485, ONVIF, third-party protocol provided by CLEVE Room, and so on; can be extended according to users’ requirements

## Abbreviations

The following abbreviations are used in this manuscript:

DE	Data Engine
IAPhome	Universal Control Platform for Smart Home
API	Application Programming Interface
SDSH	Software-defined Smart Home
SPOT	Smartphone-Based Platform for Smart-Home IoT Systems
IAP	Industrial Automation Universal Technology Platform
PLC	Programmable Logic Controller
IoT	Internet of Things
HCI	Human-computer Interaction; include HMI, Engineer Station, etc.
HMI	Human-Machine Interface
IPC	Industrial Personal Computer
IAPview	HMI Software based on IAP
IAPplant	Device-oriented Database Configuration Software based on IAP
IAPlogic	Graphical Configuration Programming Tool based on IAP
IAPdata	Data Analysis Software based on IAP
IAPbox	Communication Gateway Software based IAPhome
IAPengi	Data Engine Software based on IAP
ARM	Acorn RISC Machine
XML	Extensible Markup Language
IP	Internet Protocol
IO	Input / Output
AI	Analogy Input Component
AO	Analogy Output Component
EXTR	Data Extracting Component
DLL	Dynamic Link Library
TV	Television
TCP	Transmission Control Protocol
MAS	Multi-agent System
RGB	Red, Green and Blue

## Appendix A

Details of some smart devices mentioned in this paper.

**Table A1:** Details of some smart devices in our smart home lab.

Sensor/Device Name	Vendor/Brand	Specification/Model	Location
Lighting sensor	CLEVEROOM [39]	CRM-Light	Living room
Human motion Sensor	CLEVEROOM [39]	LH-91	Living room, Bedroom
Smart curtain	LANBOO [40]	Electric curtain, belt	Living room
Strip light	OPPLE [41]	T5	Living room
Overhead light	OPPLE [41]	T5	Bedroom, Corridor
RGB light	WEIZHILINGT [42]	Dream strip light	Bedroom
Monitoring device	HIKVISION [43]	DS-DS-2CD3120FD-IS(B)	Living room, Bedroom, Dining room, Entrance hall, Balcony
Smart mirror/ switch	CVTE [44]	M15CA-C	Entrance hall
Smart air-conditioning	DAIKIN [45]	FSSP80BA/RSQ150BAV	Living room, Bedroom
	HITACHI [46]	RCI-125HN7Q/RAS-125HNY7Q	
Electric Meter	LCKJ [47]	LCDG-DDSD-114	Kitchen
